# 

**Published:** 1846-01-01

**Authors:** 


BUFFALO

### MEDICAL JOURNAL.

EDITED BY

AUSTIN FLINT, M. D.

**Vol. 1. BUFFALO, JANUARY, 1846. No. 8.**

### CONTENTS:

### ORIGINAL COMMUNICATIONS.

Notes of an European Tour. By F. H. HAMILTON, M. D., Prof. of Surgery in Geneva Medical College. P. 170
Medical Quackery. By WM. TREAT, M. D. - - - 173
Letter from Accoucheur. No. 4. - - - 176
Needle found in the heart of a Cow. By J. H. BEECH, of Gaines, N. Y. - - - 177
Distinctive characters of Remittent, Typhoid, and Typhus Fevers. By the Editor. - - - 178

### EDITORIAL, MEDICAL INTELLIGENCE, ETC.

Water Cure—Confessions and observations of Sir Edward Lytton Bulwer. - - - 183
Summary of transactions of the College of Physicians of Philadelphia, from May to October, 1845, inclusive. 187
Introductory Lecture. By Walter Channing, M. D. - - - 189
The People of the State of New-York, vs. Wm. B. Waterman. - - - 190
Iodide of Iron. - - - 191
On the nature of the Green Alvine evacuations of Children. - - - 191
Dr. Pratt's artificial Nipple. - - - 192
The Therapeutic influence of heated atmosphere. - - - 192
Petrified Human Body. - - - 192

BUFFALO:
JEWETT, THOMAS & CO., PRINTERS AND PUBLISHERS,
Exchange Buildings, Third Story, Main-Street.

